# Supplementary material for: Structural insights into cauliflower mitoribosome in translation state and in association with a late assembly factor
Source: Nat Commun. 2025 Dec 2;16:10839. doi: 10.1038/s41467-025-65864-z (PMC12672651; doi:10.1038/s41467-025-65864-z)
Supplement: Supplementary file 1 — Supplementary Information [file 41467_2025_65864_MOESM1_ESM.pdf]

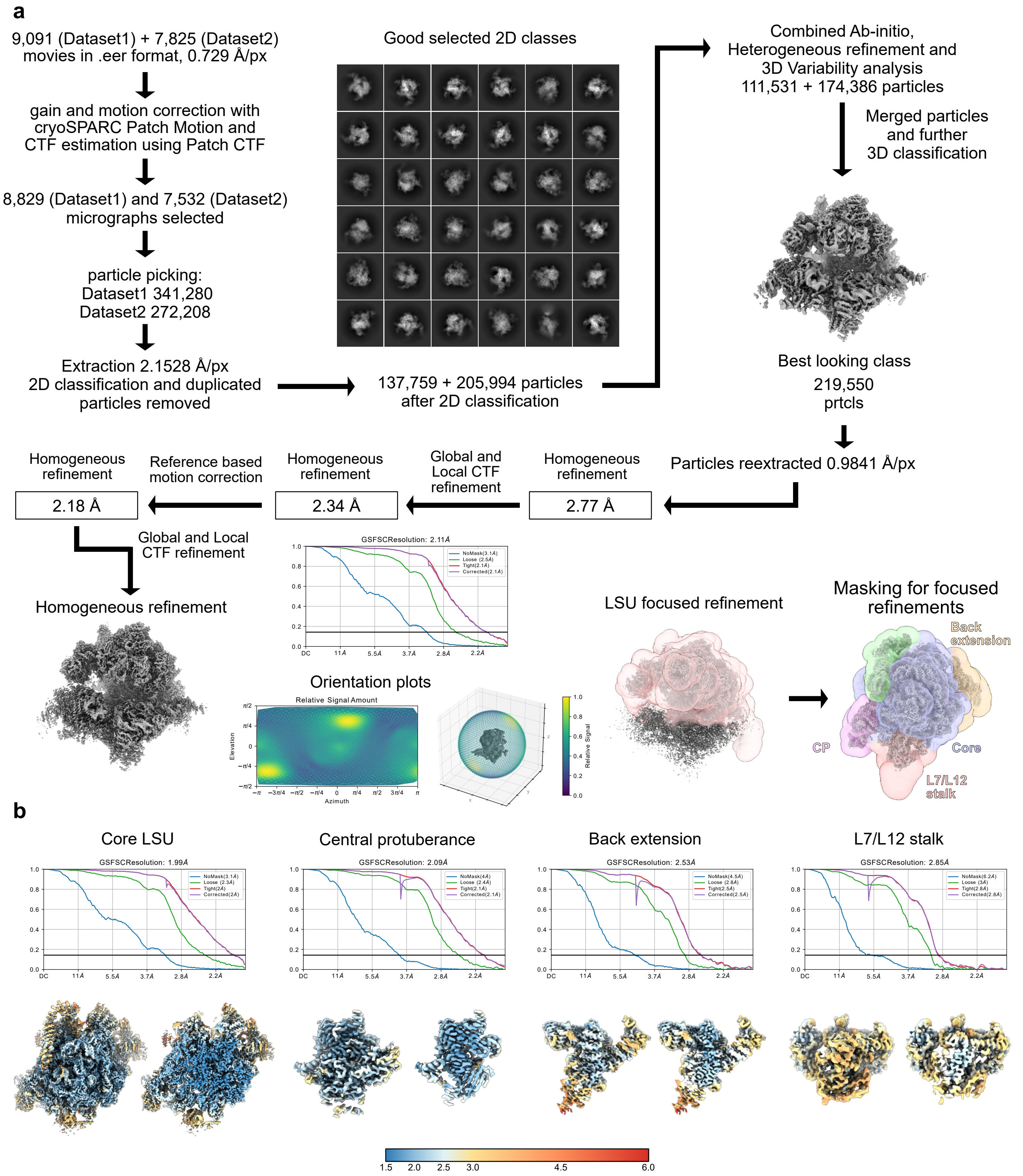

Supplementary Figure 1 : Single-particle data processing workflow of the high resolution mitoribosome

Graphical summary of the processing workflow described in Methods. **a** Post-processing, 2D and 3D classification. An orientation plot generated from the Orientation Diagnostic cryoSPARC tool is shown. **b** Focused refinements for the LSU, with all the masks used shown. For the final reconstruction, GSFSC curves are plotted, with resolution calculated at the 0.143 threshold. Local resolution plotted on the maps were generated using the built-in cryoSPARC tool with default parameters (FSC=0.143 threshold), all using the same resolution scale, with maps also shown in cut view.

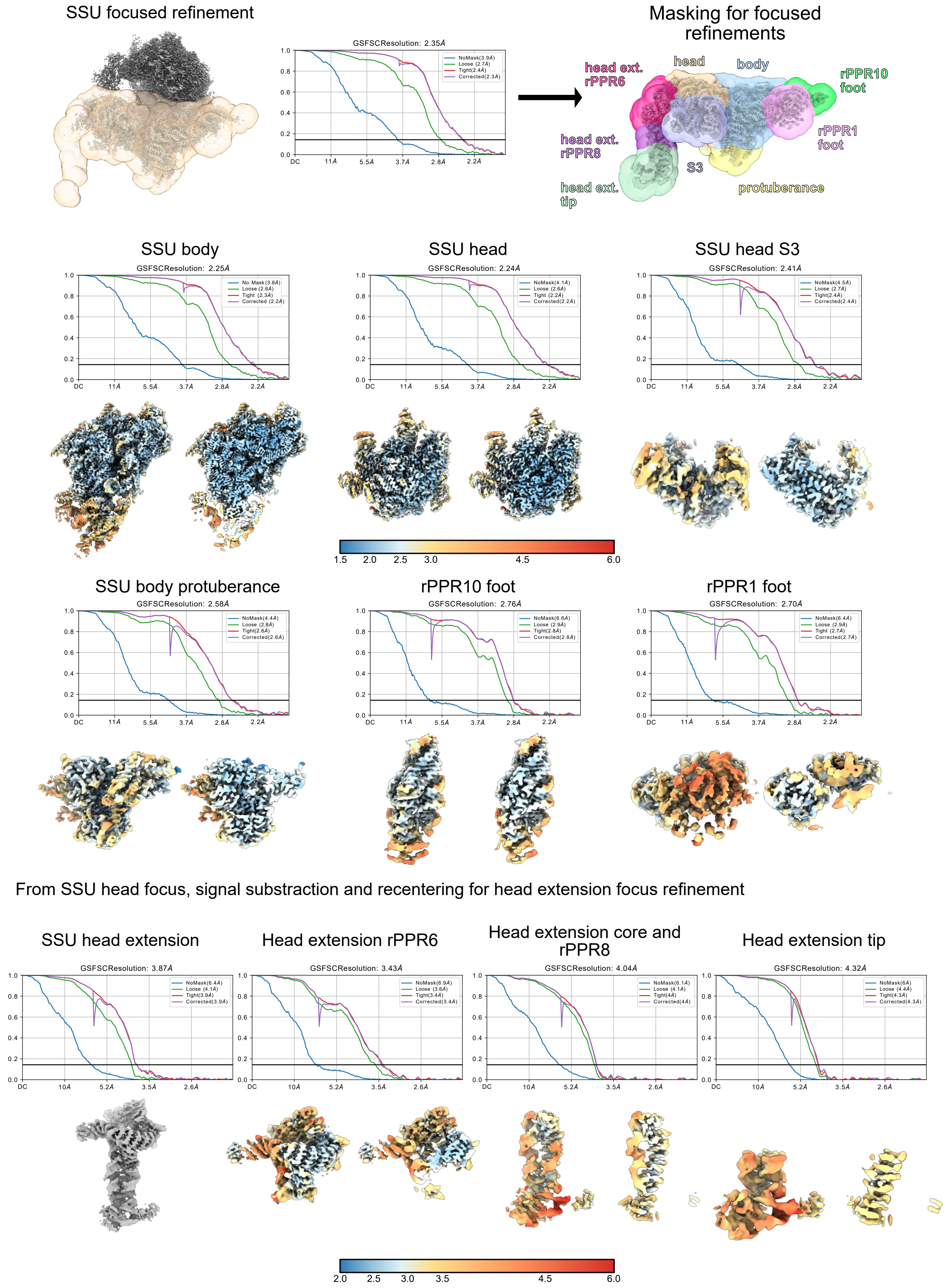

<u>Supplementary Figure 2 :</u> FSC plots of the SSU from the high resolution mitoribosome

Focused refinements for the SSU, with all the masks used shown. For the final reconstruction, GSFSC curves are plotted, with resolution calculated at the 0.143 threshold. Local resolution plotted on the maps were generated using the built-in cryoSPARC tool with default parameters (FSC=0.143 threshold), with a resolution scale different for the SSU head extension, with maps also shown in cut view.

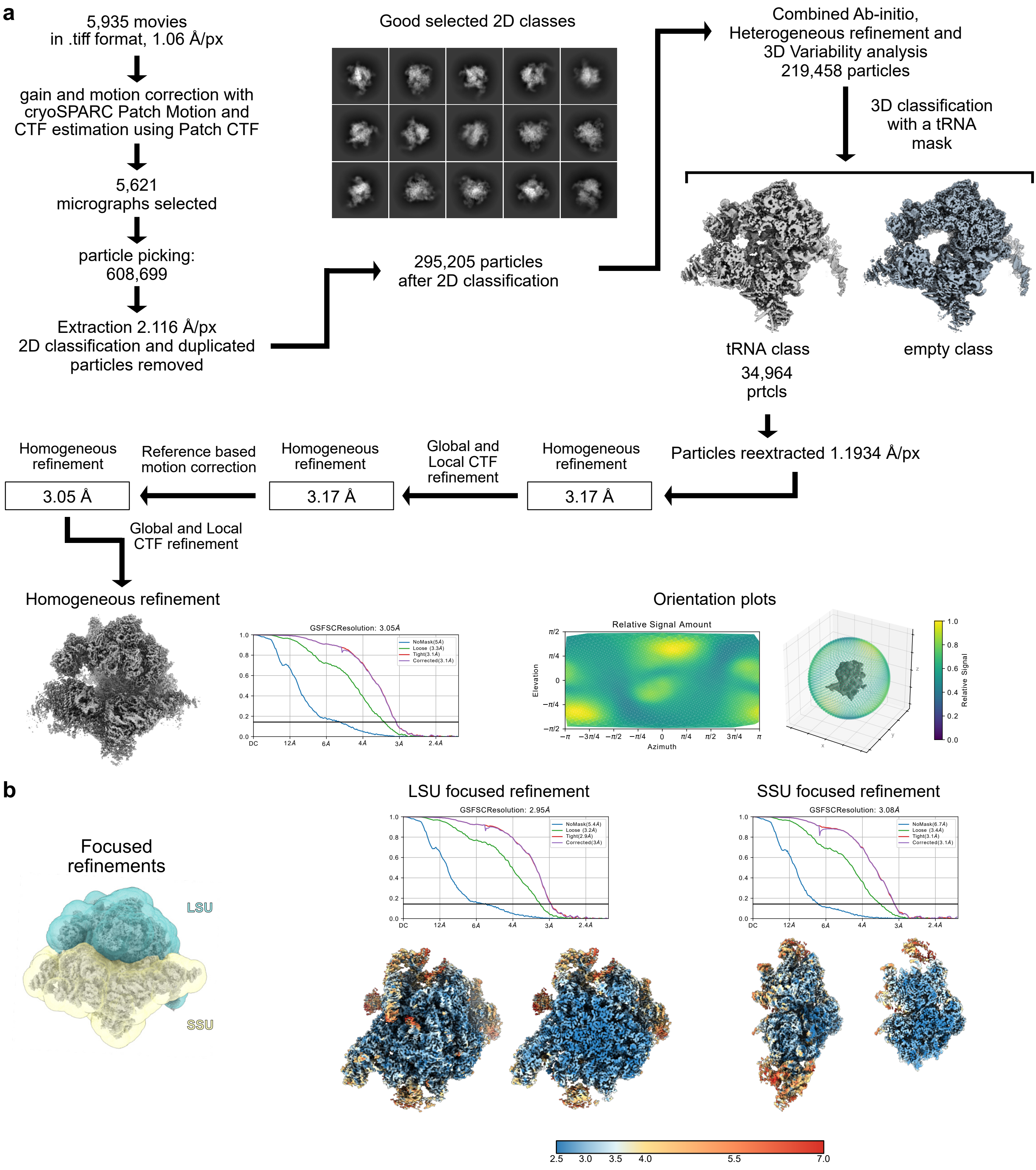

Supplementary Figure 3 : Single-particle data processing workflow of the stalled tRNA mitoribosome

Graphical summary of the processing workflow described in Methods. **a** Post-processing, 2D and 3D classification. An orientation plot generated from the Orientation Diagnostic cryoSPARC tool is shown. **b** Focused refinements for the tRNA class, with all the masks used shown. For the final reconstruction, GSFSC curves are plotted, with resolution calculated at the 0.143 threshold. Local resolution plotted on the maps were generated using the built-in cryoSPARC tool with default parameters (FSC=0. 143 threshold), all using the same resolution scale, with maps also shown in cut view.

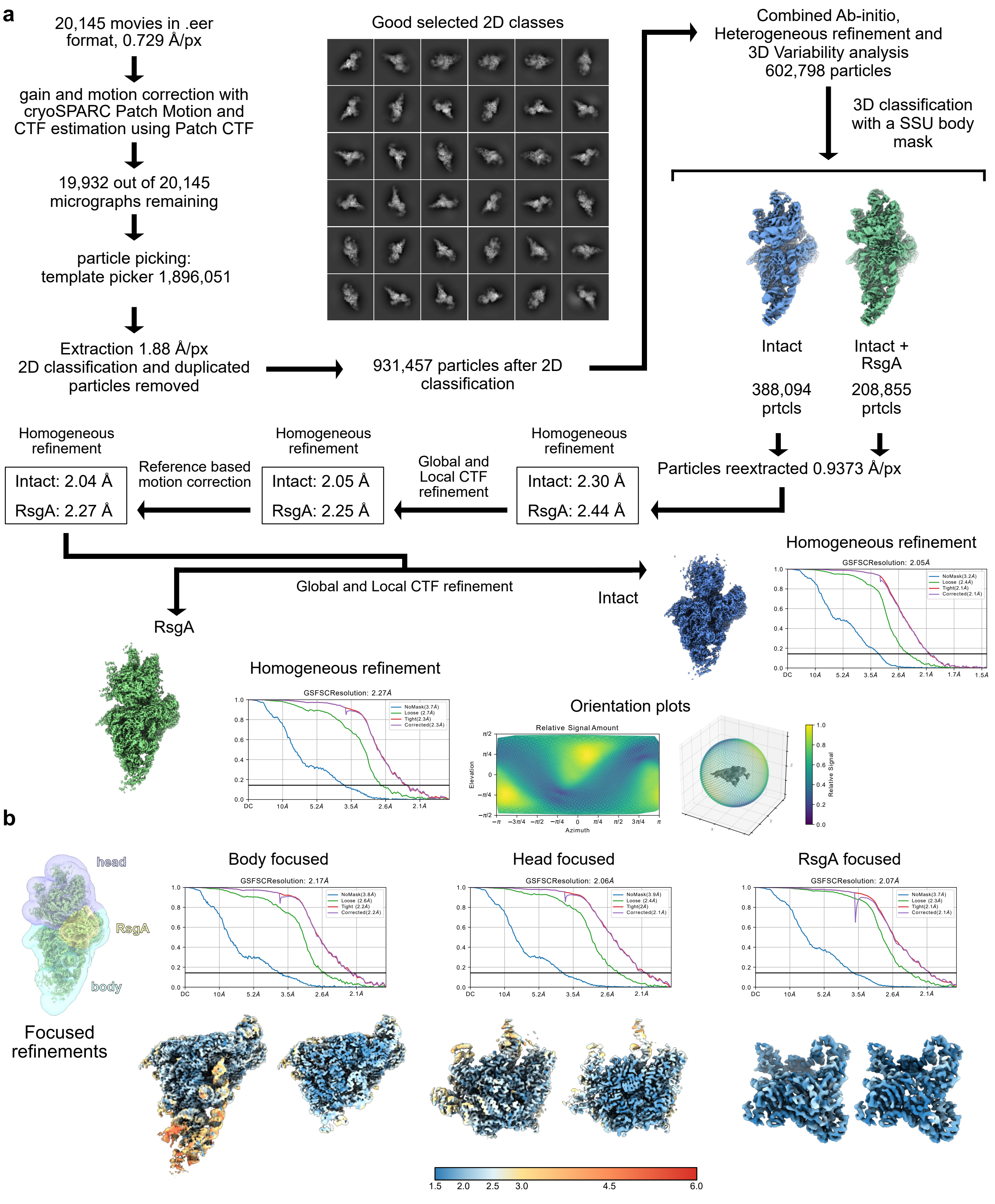

Supplementary Figure 4 : Single-particle data processing workflow of the SSU with RsgA

Graphical summary of the processing workflow described in Methods. **a** Post-processing, 2D and 3D classification. An orientation plot generated from the Orientation Diagnostic cryoSPARC tool is shown. **b** Focused refinements for the RsgA class, with all the masks used shown. For the final reconstruction, GSFSC curves are plotted, with resolution calculated at the 0.143 threshold. Local resolution plotted on the maps were generated using the built-in cryoSPARC tool with default parameters (FSC=0.143 threshold), all using the same resolution scale, with maps also shown in cut view.

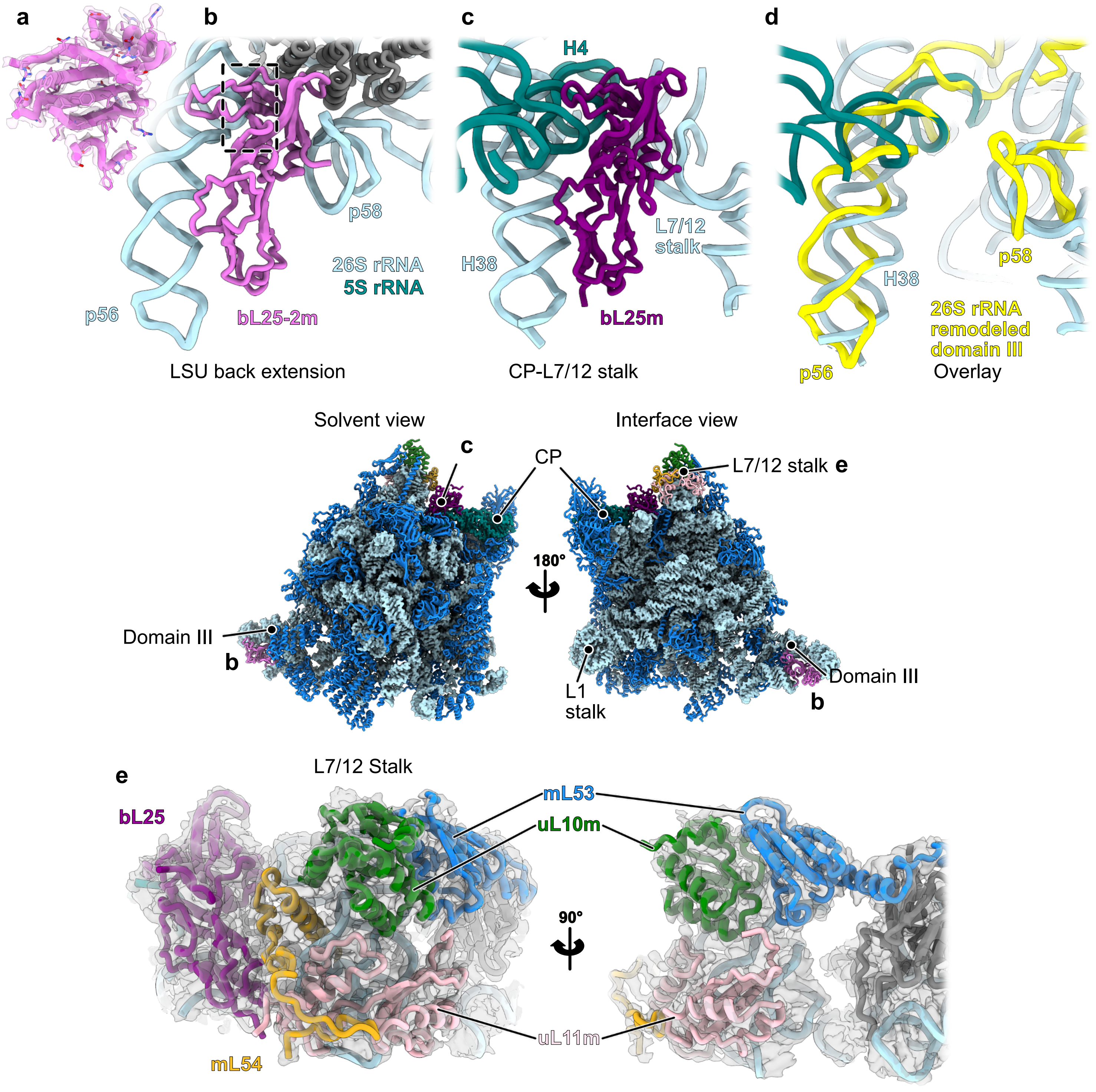

Supplementary Figure 5 : Newly resolved r-proteins in the LSU

a-d Newly identified r-protein bL25-2m in the LSU back extension, remodeling the 26S domain III. **a** show a close-up of bL25-2m, shown in **b**, with the experimental density. **c** Close-up view of the canonical bL25m r-protein in the mitoribosome. **d** Overlay of the remodeled domain III from **b**, shown in yellow, with the 5S and 26S rRNA from **c** showing the structural mimicry of the rRNA, with the base of p56 mimicking H4 of the 5S and p56's tip mimicking H38, as well as p58 mimicking the base of the L7/17 stalk. **e** View of the L7/12 stalk with the experimental density showing mL54 which was previously unresolved. The different areas of the LSU are shown in the middle of the figure, with panel **b**, **c** and **e** positions indicated.

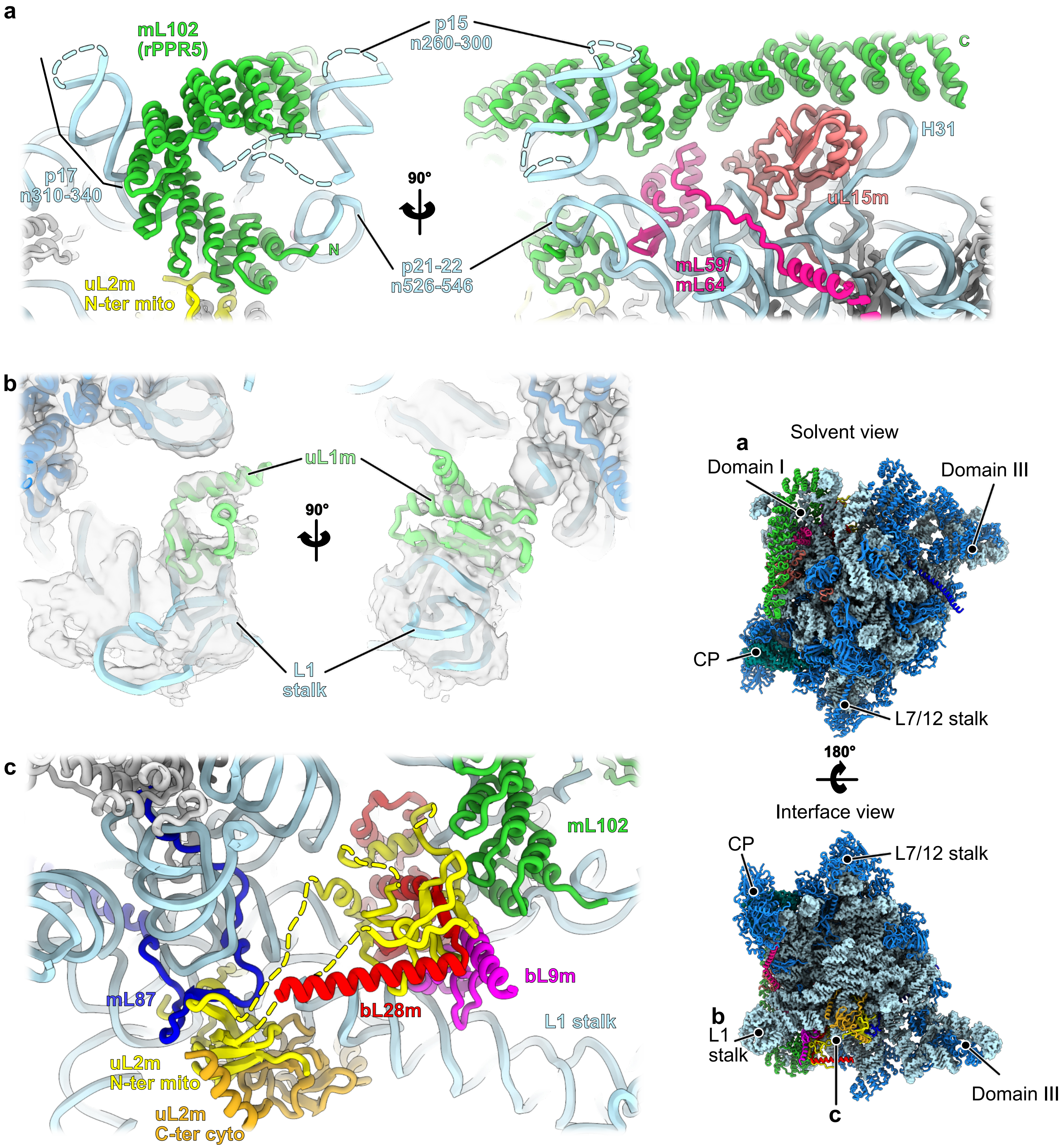

Supplementary Figure 6 : Improved model in the LSU

a Close-up view of the mL102 area. The rPPR protein mL102 interacts with three r-proteins, uL15m, mL59/64 and uL2m (N-ter mito). The N-terminal part of the protein largely interacts with rRNA and contributes to the remodeling of domain I, forming p15, p17 and p21-22, specific helices of the plant mitoribosome. Unmodeled densities of the rRNA are shown are blue dashed lines. **b** View of the L1 stalk at low threshold, revealing the presence of the uL1m protein. **c** In the plant mitoribosome, uL2m is split into two parts. What would correspond to the N-terminal part of the canonical uL2 is encoded as uL2m in the mitochondrial genome (yellow), whereas the C-terminal part is encoded in the nuclear genome (orange). The uL2m N-ter mito possesses large insertions that extend away from the uL2 core to make intricate interactions with bL28m, bL9m and mL102. Close to the uL2 core it also makes direct interactions with mL87. The different areas of the LSU are shown on the right hand-side of the figure, with panel **a**, **b** and **c** positions indicated.

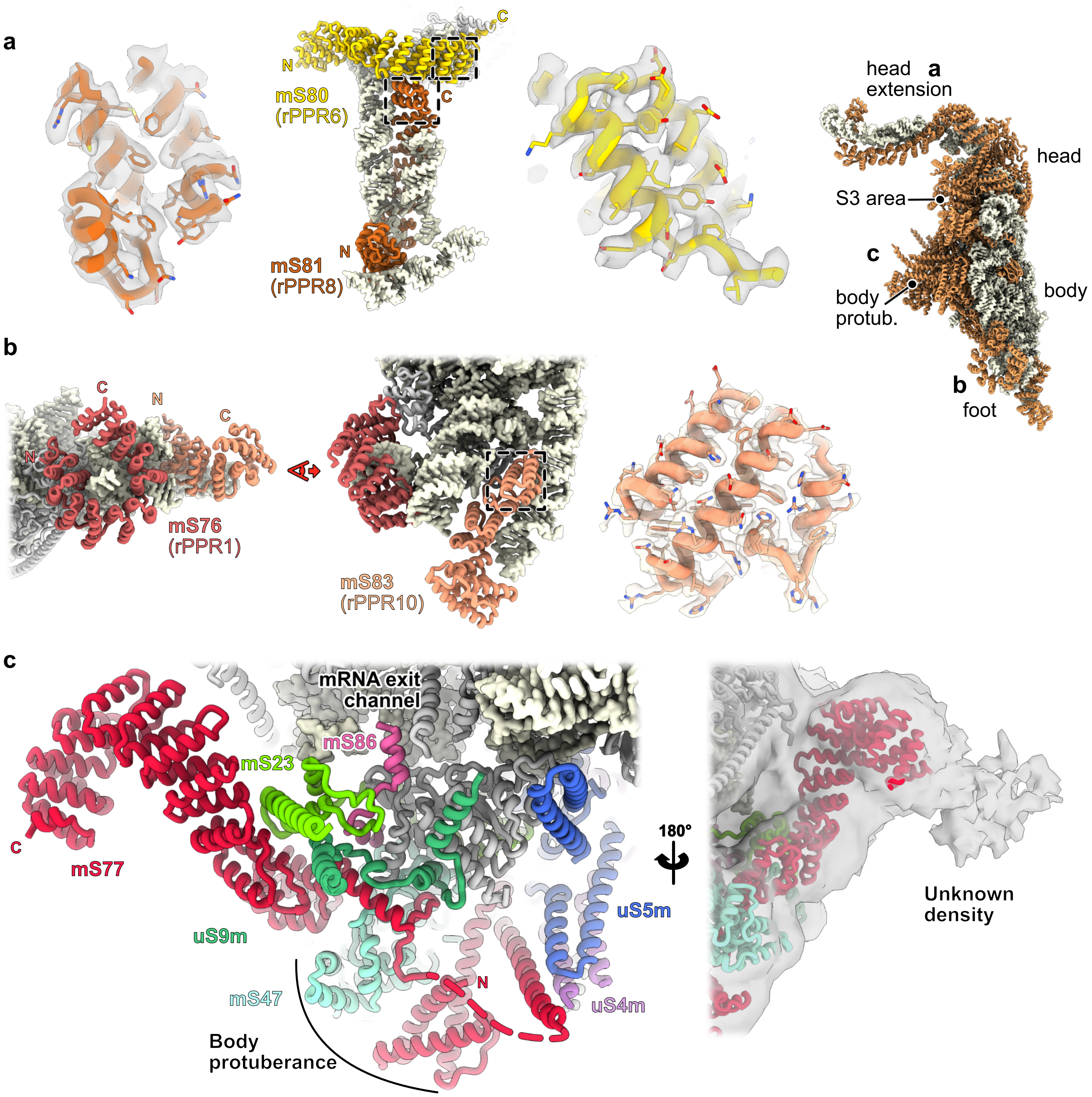

Supplementary Figure 7 : PPR proteins of the SSU

a On the SSU head extension, two PPR proteins are found, mS80 and mS81. Portions of the atomic models (highlighted by dashed boxes) are shown in the experimental density. **b** At the foot of the SSU, two PPR proteins are found, m76 and mS83. Portions of the atomic models (highlighted by dashed boxes) are shown in the experimental density. **c** The newly identified r-protein mS77 (red) spans from the SSU body protuberance to the exit mRNA channel. It is composed of two main domains. Forming part of the SSU body protuberance, the N-terminal part interacts with extensions of uS5m and uS4m as well as mS47. Through a flexible portion of the protein (represented by dashed-lines) it wraps around the SSU body and its C-terminal part is composed of a long stretch of PPR repeats, interacting with mS23 and an extension of uS9m. At the mRNA exit channel a small portion of mS86 is also visible, sandwiched between mS23 and uS2m. At lower threshold, we also observe additional densities at the C-terminus of mS77, that could indicate that it serves as a binding platform for additional factors. C- and N-termini of the proteins of interest are indicated by colored N and C. The different areas of the SSU are shown on the right hand-side of the figure, with panel **a**, **b** and **c** positions indicated.

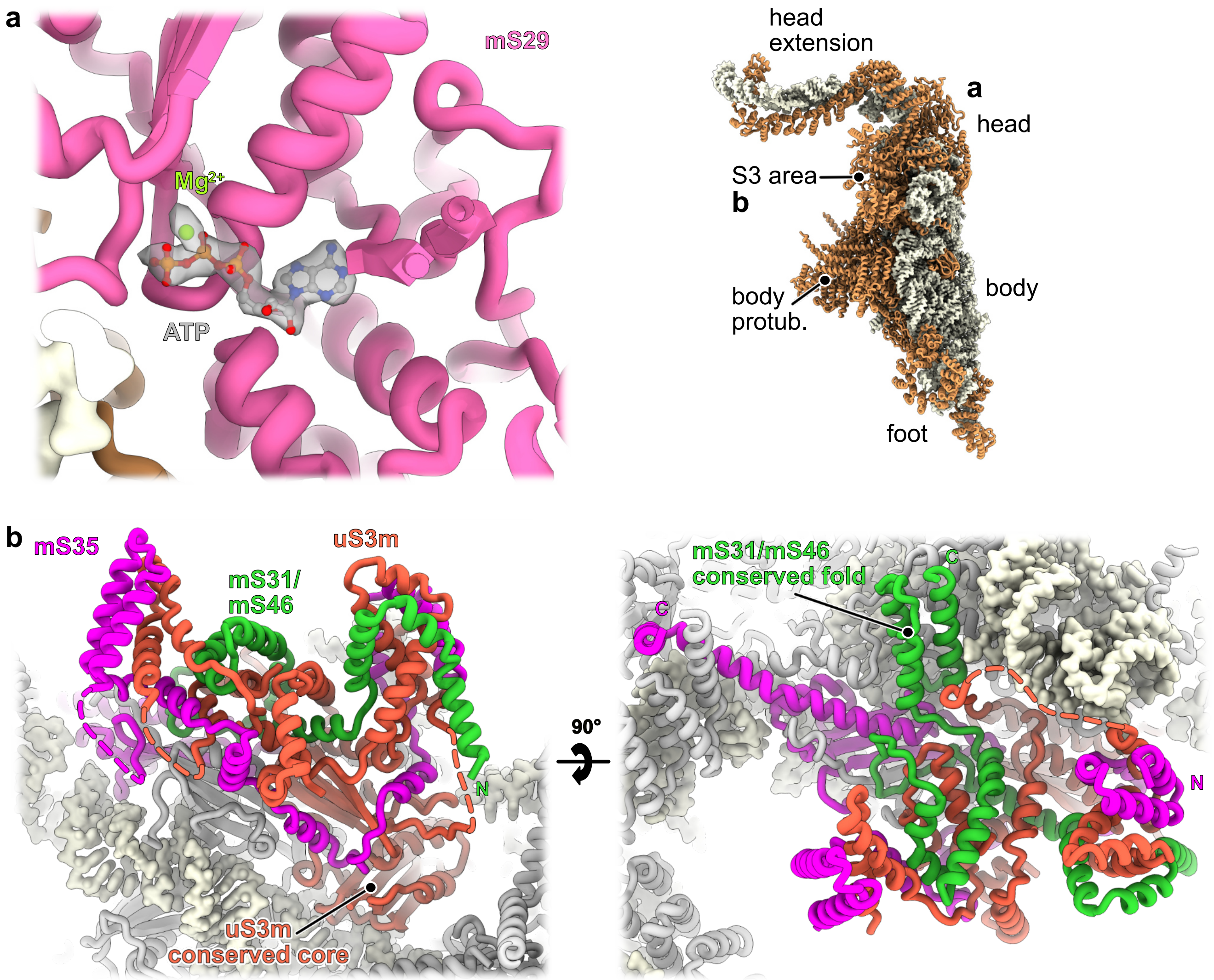

Supplementary Figure 8 : Improved model in the SSU

a Close-up view of mS29 on the SSU head, showing the ATP with Mg^{2+} in its density. **b** View of the SSU head protuberance, or S3 area. This domain is formed by a large insertion in uS3m, forming intricate interactions with the N-terminal part of mS35 and a large portion of mS31/mS46. with C- and N-termini of the proteins of interest are indicated by colored N and C. The different areas of the SSU are shown on the right hand-side of the figure, with panel **a** and **b** positions indicated.

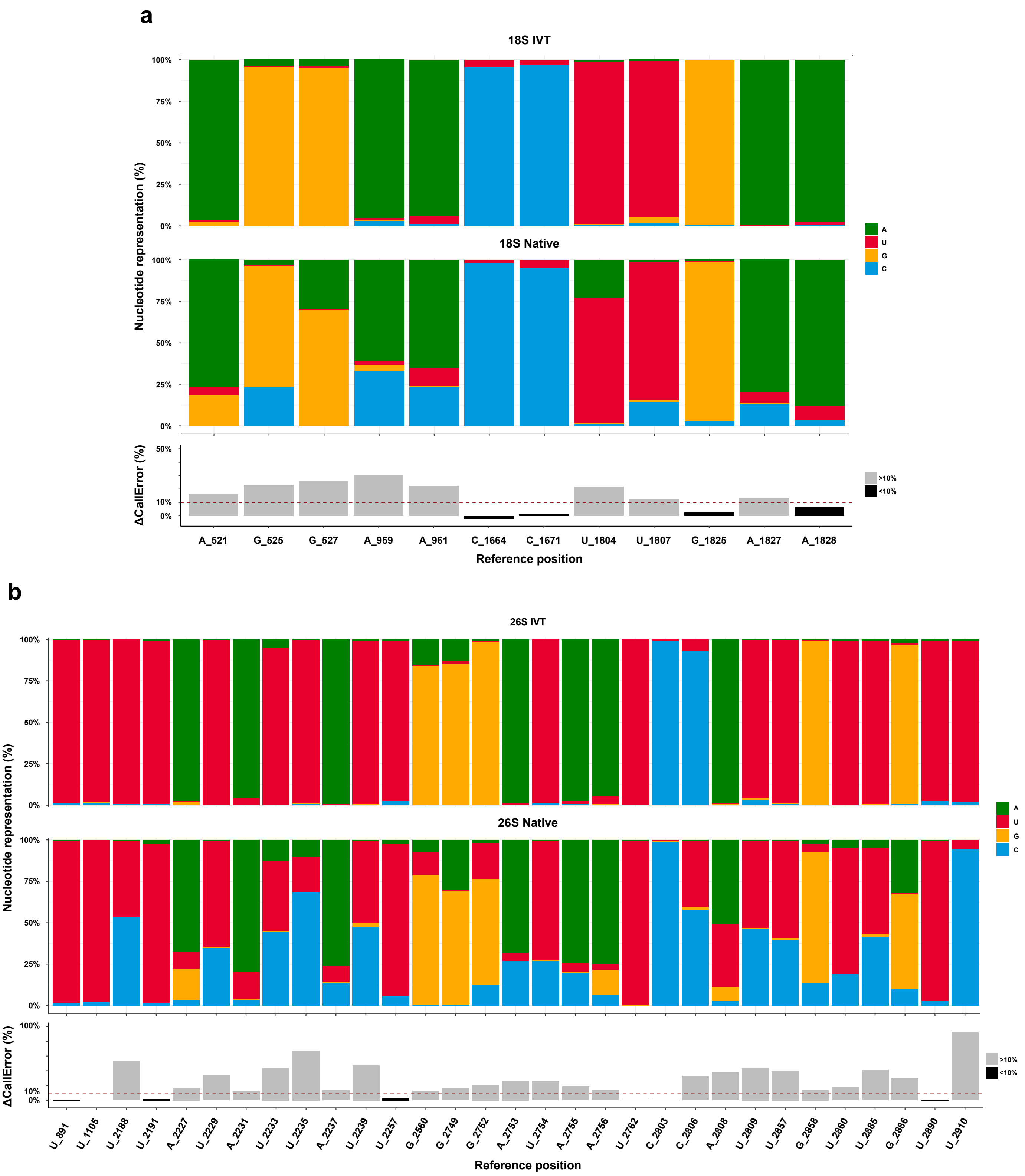

<u>Supplementary Figure 9:</u> Nucleotide frequency at positions of interest

Base-called nucleotides and their percentage presented for all positions of interest of 18S rRNA (**a**) and 26S rRNA (**b**). Comparison of IVT and Native rRNAs is presented, with the latter displaying base-calling errors accompanying the presence of putative rRNA modifications. Difference in Native versus IVT base-calling error (ΔCallError, %), is calculated for each base and defined as Error Native (%) - Error IVT (%), where the error corresponds to the highest non reference base observed in Native data at that position. Bases exhibiting ΔCallError above 10% are considered positions of interest. Positions with identified modifications from the cryo-EM map and mass spectrometry are also included. Base-called nucleotides are colored. Color code corresponds to the four bases of the rRNA sequence, Green, A; Red, U; Orange, G; Blue, C. The kmer 26S: 2752-2756 was assigned to the modification D2754.

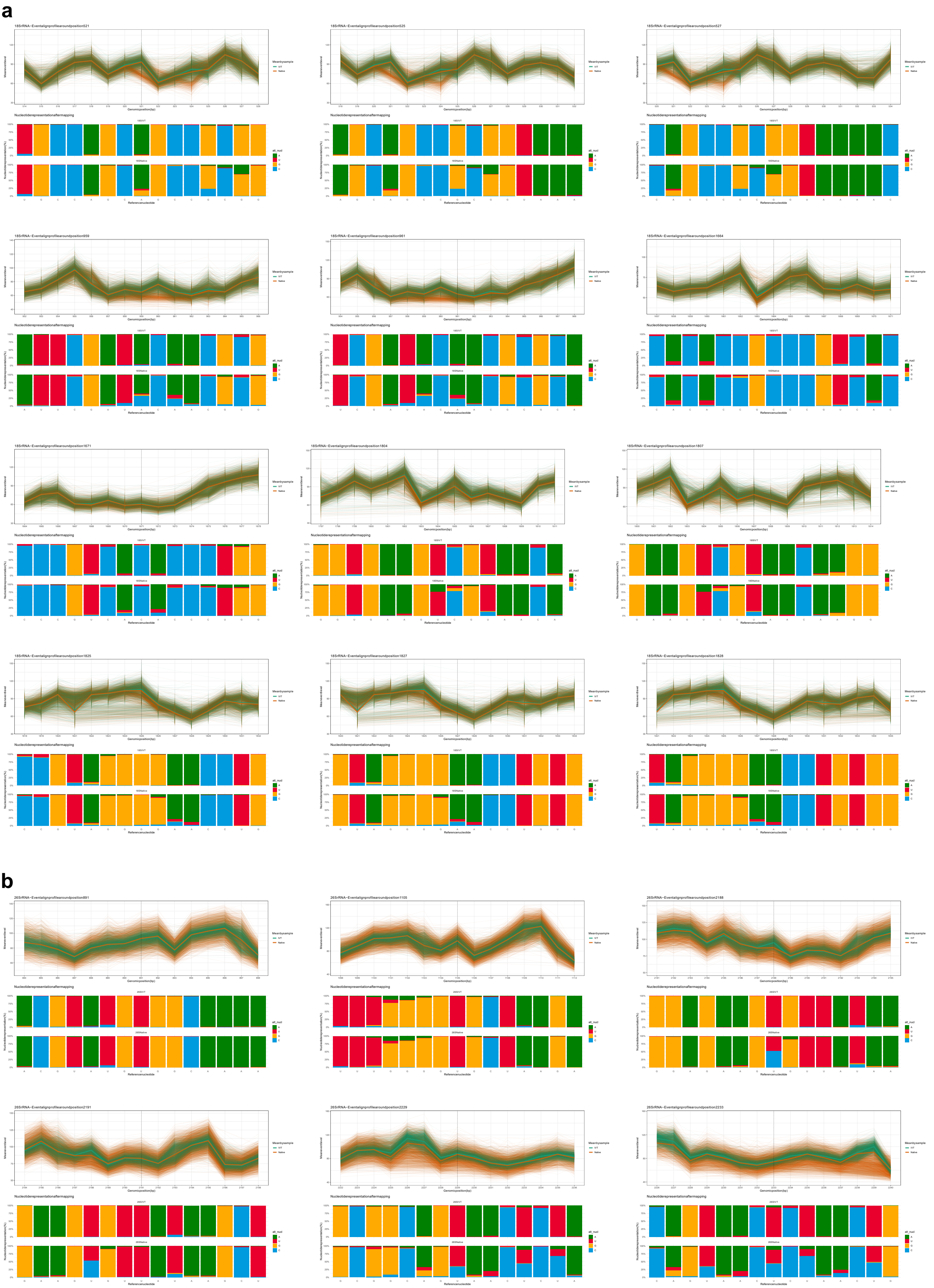
a
18SrRNA−Eventalignprofilearoundposition521
18SrRNA−Eventalignprofilearoundposition525
18SrRNA−Eventalignprofilearoundposition527
18SrRNA−Eventalignprofilearoundposition959
18SrRNA−Eventalignprofilearoundposition961
18SrRNA−Eventalignprofilearoundposition1664
18SrRNA−Eventalignprofilearoundposition1671
18SrRNA−Eventalignprofilearoundposition1804
18SrRNA−Eventalignprofilearoundposition1807
18SrRNA−Eventalignprofilearoundposition1825
18SrRNA−Eventalignprofilearoundposition1827
18SrRNA−Eventalignprofilearoundposition1828
b
26SrRNA−Eventalignprofilearoundposition891
26SrRNA−Eventalignprofilearoundposition1105
26SrRNA−Eventalignprofilearoundposition2188
26SrRNA−Eventalignprofilearoundposition2191
26SrRNA−Eventalignprofilearoundposition2229
26SrRNA−Eventalignprofilearoundposition2233
Nucleotiderepresentationaftermapping
Genomicposition(bp)
Referencenucleotide
Meanbysample
IVT
Native
alt_nucl

b cont

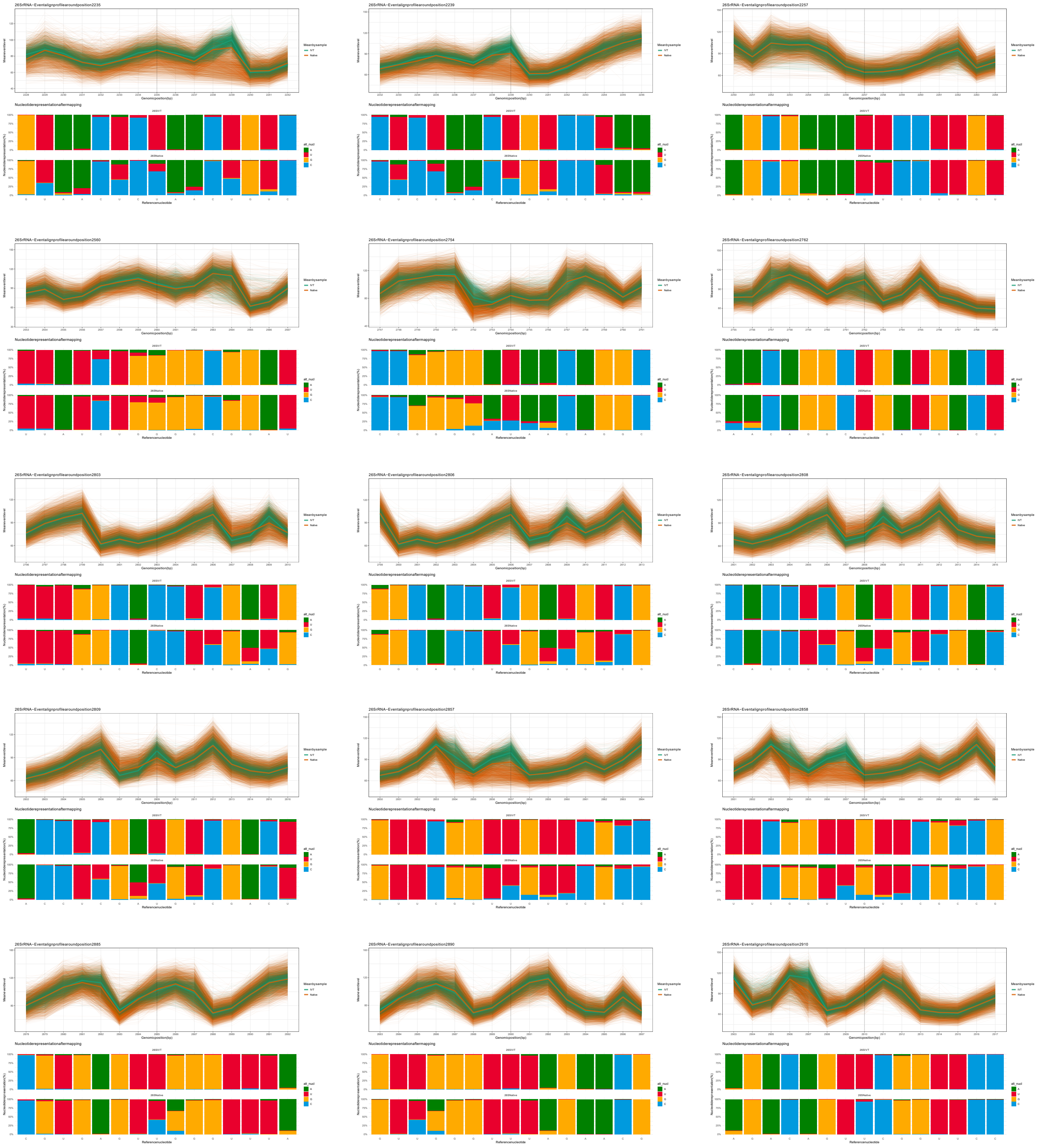

<u>Supplementary Figure 10</u>: Current intensity and nucleotide frequency at positions of interest

Per-read current intensity analysis and nucleotide frequency in the 15-mer regions surrounding the sites of interest of 18S rRNA (**a**) and 26S rRNA (**b**). Green lines correspond to native reads whereas brown lines to IVT reads. Thick green and brown lines represent the mean current intensity of native and IVT reads, respectively.

a

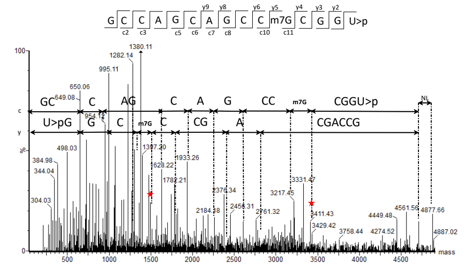

m/z	Charge state	Calculated mass	Sequence	RNase	Position
1218.65	4	4877.6	GCCAGCAGCC[m7G]CGGU>p	RnaseH (1,2)/ Rnase4	A521/m7G525/G527

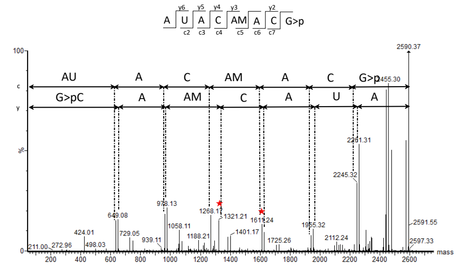

m/z	Charge state	Calculated mass	Sequence	RNase	Position
1294.67	2	2590.3	AUAC[AM]ACG>p	RnaseH (3,4)/ RNAseT1	A959/AM961

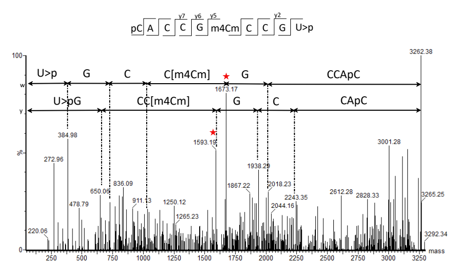

m/z	Charge state	Calculated mass	Sequence	RNase	Position
1086.8	3	3262.4	pCACCG[m4Cm]CCGU>p	RnaseH (5,6)/ RNase4	m4Cm1664

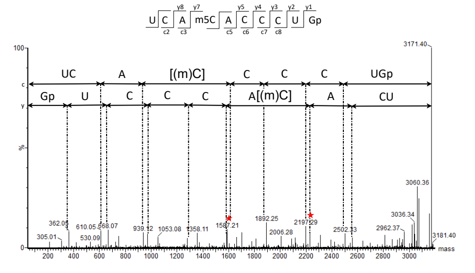

m/z	Charge state	Calculated mass	Sequence	RNase	Position
1056.46	3	3171.4	UCA[CM]ACCCUGp	RnaseH (5,6)/ RNaseT1	(m)C1671

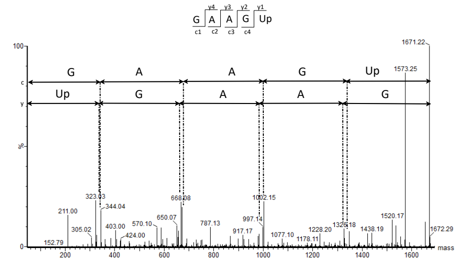

m/z	Charge state	Calculated mass	Sequence	RNase	Position
835.11	2	1671.2	GAAGUp	RnaseH (6)/ RNaseA	U1804

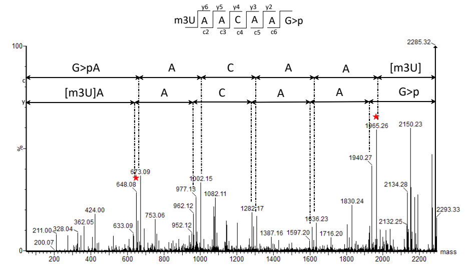

m/z	Charge state	Calculated mass	Sequence	RNase	Position
1142.16	2	2285.3	[m3U]AACAAG>p	RnaseH (6)/ RNaseT1	m3U1807

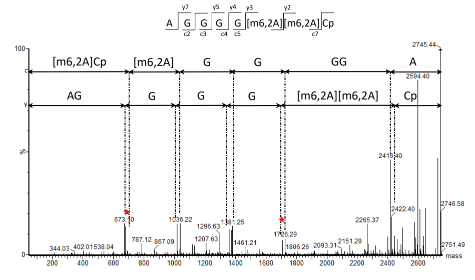

m/z	Charge state	Calculated mass	Sequence	RNase	Position
1372.21	2	2745.4	AGGGG[m6,2A][m6,2A]Cp	RnaseH (6)/ RnaseA	G1825/m62A1827/ m62A1828

b

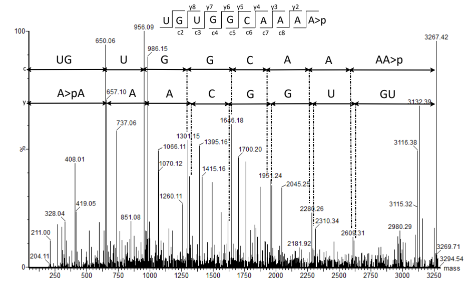

m/z	Charge state	Calculated mass	Sequence	RNase	Position
1088.47	3	3267.4	UGUGGCAAAA>p	RnaseH (1,2)/ RnaseMC1	U891

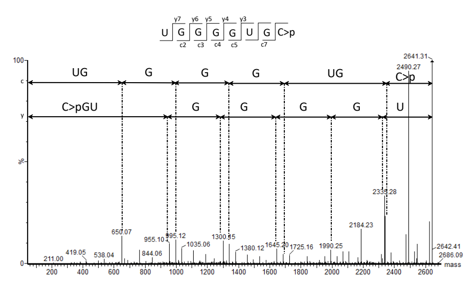

m/z	Charge state	Calculated mass	Sequence	RNase	Position
1320.15	2	2641.3	UGGGGUGC>p	RnaseH (3,4)/ RnaseMC1	U1105

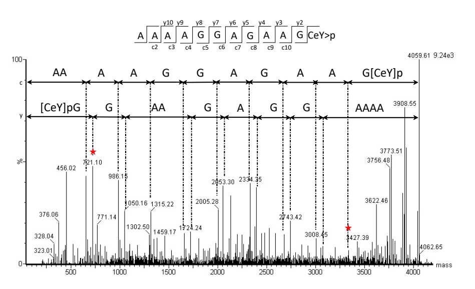

m/z	Charge state	Calculated mass	Sequence	RNase	Position
1352.52	3	4059.6	AAAAGGAGAAG[CeY]p	RnaseH (5,6)/ RnaseA	Ψ2188

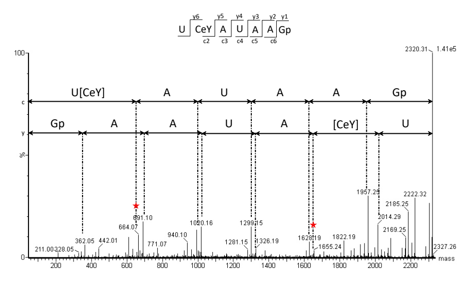

m/z	Charge state	Calculated mass	Sequence	RNase	Position
1159.66	2	2320.3	U[CeY]AUAAGp	RnaseH (5,6)/ RnaseT1	Ψ2191

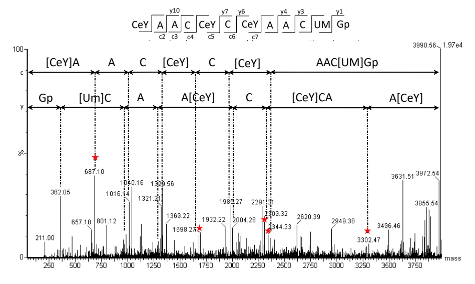

m/z	Charge state	Calculated mass	Sequence	RNase	Position
1329.51	3	3990.5	[CeY]AAC[CeY]C[CeY]AAC[UM]Gp	RnaseH (5,6)/ RnaseT1	Ψ2229/Ψ2233/ Ψ2235/UM2239

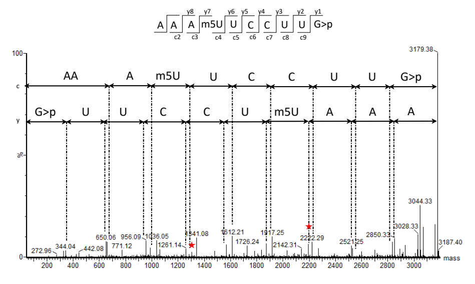

m/z	Charge state	Calculated mass	Sequence	RNase	Position
1059.11	3	3179.3	AAA[m5U]UCCUUG>p	RnaseH (5,6)/ RnaseT1	m5U2257

b cont.

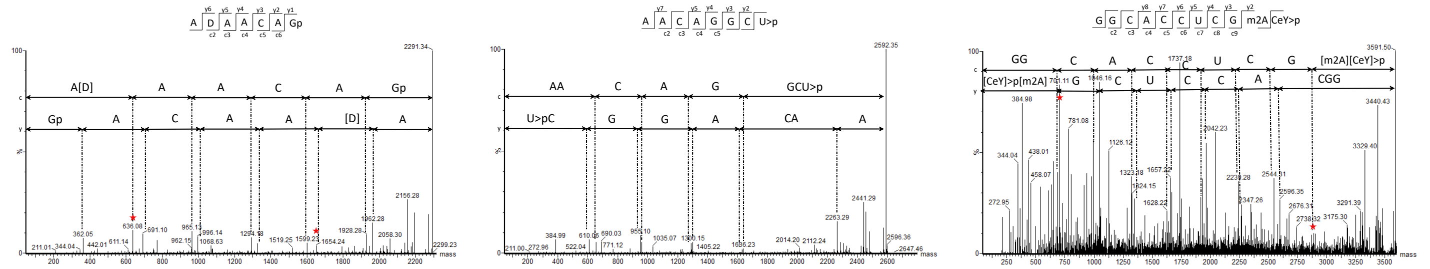

m/z	Charge state	Calculated mass	Sequence	RNase	Position
1145.16	2	2291.3	A[D]AACAGp	RnaseH (7,8)/ RnaseT1	D2754

m/z	Charge state	Calculated mass	Sequence	RNase	Position
1295.67	2	2592.3	AACAGGCU>p	RnaseH (7,8)/ Rnase4	U2762

m/z	Charge state	Calculated mass	Sequence	RNase	Position
1196.49	3	3591.5	GGCACCUCG[m2A][CeY]>p	RnaseH (7,8)/ Rnase4	C2803/C2806/ m2A2808/Ψ2809

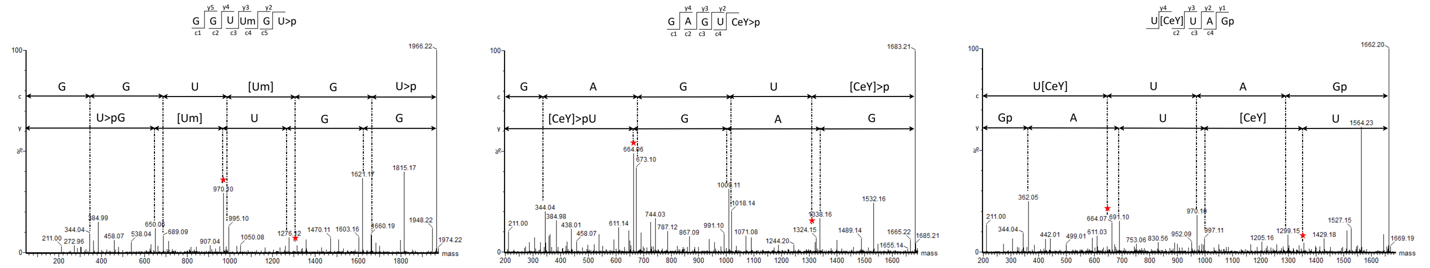

m/z	Charge state	Calculated mass	Sequence	RNase	Position
982.6	2	1966.2	GGU[Um]GU>p	RnaseH (8,9)/ Rnase Cus	Um2857/G2858

m/z	Charge state	Calculated mass	Sequence	RNase	Position
841.1	2	1683.2	GAGU[CeY]>p	RnaseH (8,9)/ Rnase4	Ψ2885

m/z	Charge state	Calculated mass	Sequence	RNase	Position
830.6	2	1662.2	U[CeY]UAGp	RnaseH (8,9)/ RnaseT1	Ψ2890

<u>Supplementary Figure 11</u>: Mass spectrometry analysis of modified nucleotides.

MS/MS spectra of modified nucleotides for 18S (**a**) and 26S rRNA (**b**). Information regarding the mass to charge ratio (*m/z*), charge state, calculated mass, sequence, RNase enzymes (including the respective oligonucleotides used for RNase H in parentheses), and positions are presented for each spectrum. "Ce" next to RNase indicates the cyanoethylation of the sample, which allows detection of Ψ. In the sequence, "CeY" corresponds to cyanoethylated Ψ. Peaks corresponding to modified signals are indicated by a star.

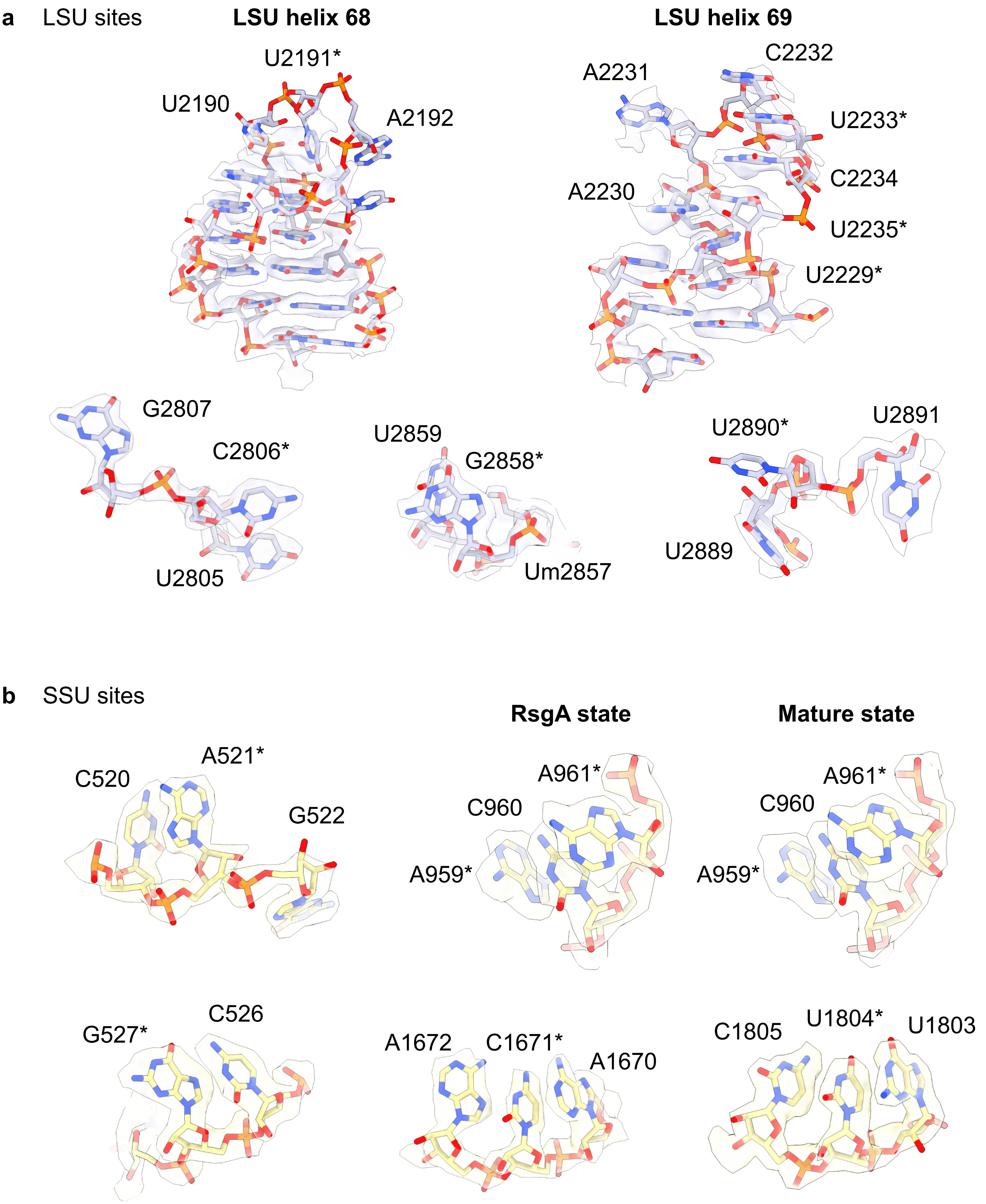

<u>Supplementary Figure 12 :</u> rRNA modifications not visible or not confirmed by cryo-EM

Close-up views of the rRNA bases in **a** the LSU and **b** the SSU where nanopore sequencing or MS predict a base modification (marked with a *). **a** in the LSU several pseudo-uridines sites are ambiguous because the densities are not resolved enough or the areas are disordered, e.g H68 and H69 as well as U2890 where the base is flipped out of the helix. **b** in the SSU, the resolution is good enough for the characterization of all possible modifications. Still, at position 961, both nanopore and MS identify a potential (m)A, but in the cryo-EM the base is seen as an A both in the RsgA and mature state.

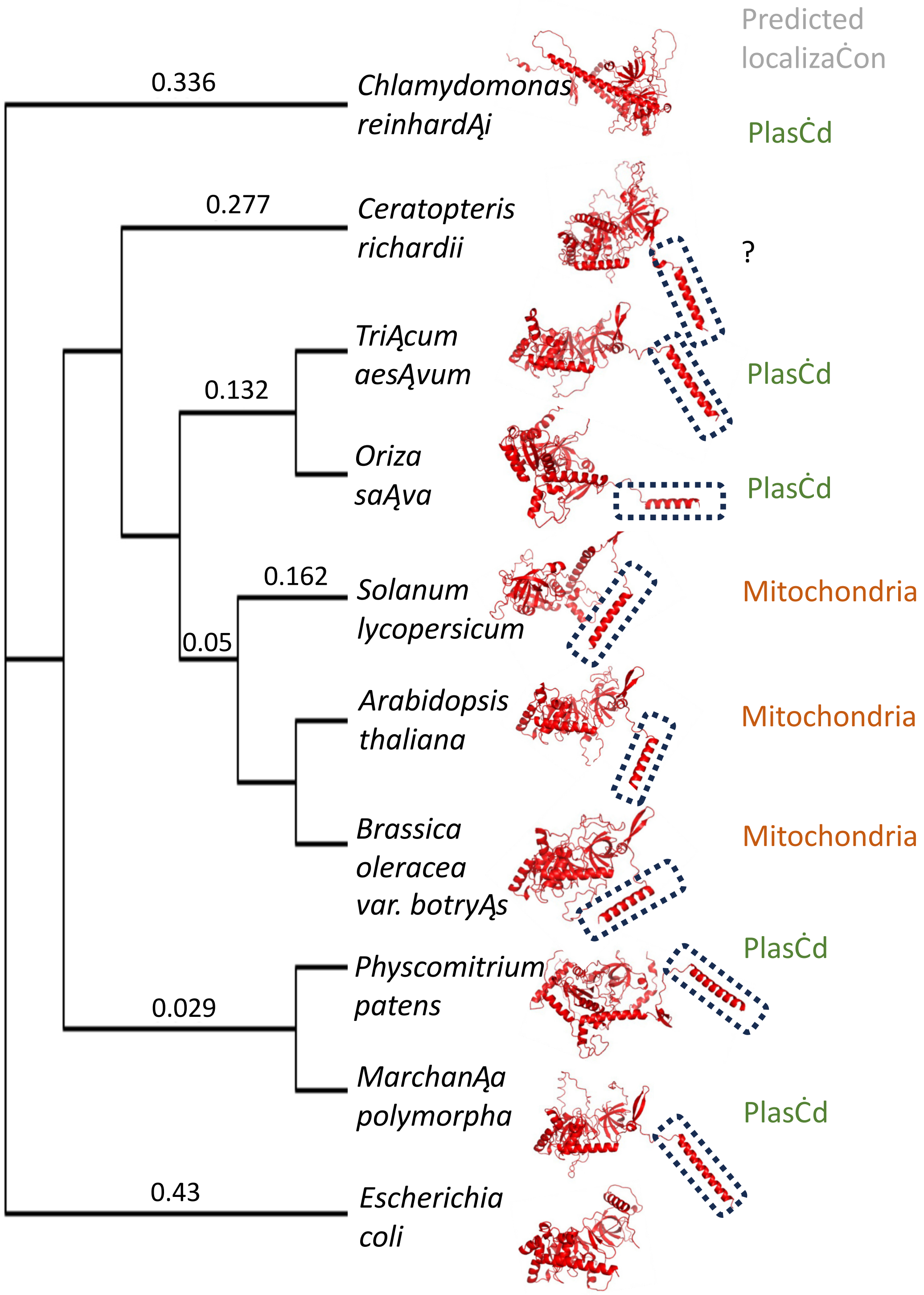

Supplementary Figure 13 : Phylogeny analysis of plant RsgA orthologs.

RsgA proteins representative from the green lineage, including algae (Chlamydomonas), fern (Ceratopteris), moss (Physcomitrium), liverwort (Marchantia) and angiosperms belonging to the group of monocotyledone plants (Triticum and Oriza) as well as dicotyledon plants (Solanum, Arabidopsis and Brassica) were compared with *E.coli* RsgA. Plant specific C-terminal extensions are highlighted by dotted line boxes. The localization predictions of proteins were obtained with TargetP (https://services.healthtech.dtu.dk/services/TargetP-2.0) and indicated on the right.

	Full high res. (PDB 9GYT - EMD-51718)	Full with tRNA (PDB 9EVS)	SSU with RsgA (PDB 9EVT)
EMDB	Unfocused EMD-51703	Unfocused EMD-50011	Unfocused EMD-50014
**Data collection and processing**			
Magnification	165,000x	130,000x	165,000x
Voltage (kV)	300	300	300
Electron exposure (e–/Å^2)	41.66	46	50.96
Defocus range (μm)	-0.5 to -2.5	-0.5 to -2.0	-0.5 to -2.5
Pixel size (Å)	0.729	1.058	0.729
Symmetry imposed	C1	C1	C1
Initial particle images (no.)	613,488	141,939	1,896,051
Final particle images (no.)	219,550	83,443	208,855
Map resolution (Å) - FSC 0.143	2.11	3.05	2.27
Map resolution range (Å)	1.6 - 4.8*	2.3 - 6.9*	1.6 - 5.1*
**Refinement**			
Initial model used (PDB code)	AF2 - 6XYW	AF2 - 6XYW	AF2 - 6XYW
FSC model (0.143)	2.1	3.0	2.3
CC Model vs. Data (mask)	0.71	0.81	0.75
Map sharpening *B* factor (Å^2)	-26.4	-36.5	-39.8
Model composition			
Non-hydrogen atoms	233460	211448	85751
Residues: Protein - Nucleotide	15010 - 4806	13711 - 4713	6452 - 1560
Water	10047	-	-
Ligands	ZN: 2<br>K: 84<br>MG: 349<br>ATP: 1	ZN: 2<br>K: 74<br>MG: 357<br>ATP: 1<br>CLM:1	ZN: 1<br>K: 10<br>MG: 85<br>ATP: 1<br>GTP: 1
B factors (Å^2)			
Protein	0.00/1098.4/68.25	0.00/283.77/67.44	0.00/274.51/53.60
Nucleotide	0.00/440.22/57.04	1.69/341.90/82.12	0.00/226.44/51.37
Ligand	18.37/90.34/48.08	3.78/141.34/41.08	12.44/76.98/37.00
R.m.s. deviations			
Bond lengths (Å)	0.006	0.007	0.004
Bond angles (°)	0.994	0.989	0.965
Validation			
MolProbity score	1.50	1.39	1.45
Clashscore	5.73	3.53	5.37
Poor rotamers (%)	0.07	0.03	0.04
Ramachandran plot			
Favored (%)	96.91	96.31	97.12
Allowed (%)	2.95	3.60	2.80
Disallowed (%)	0.15	0.09	0.08

* min to 75th in cryoSPARC Local Resolution Estimation (FSC 0.143)

Supplementary Table 1 : cryo-EM model statistics

Name of the protein	Arabidopsis UNIPROT	TAIR ID Arabidopsis	Modeled protein (Brassica oleracea var.botrytis)	ChainID	Location
uL1m	Q8RWT4	At2g42710	GWHPBJSH045905 (not modeled but visible)	A	L1 stalk
uL2m C-ter	Q8VZU4	At2g44065	GWHPBJSH030998	B	
uL2m N-ter mito	P93311	AtMg00560	GWHPBJSH056804	C	
uL3m	Q9LRN8	At3g17465	GWHPBJSH008303	D	
uL4m	Q8VY61	At2g20060	GWHPBJSH075117	E	
uL5m	P42793	AtMg00210	GWHPBJSH000742	F	Central Protuberance
uL6m	Q9ZPX2	At2g18400	GWHPBJSH074602	G	
bL9m	Q9LVU5	At5g53070	GWHPDUBS006976	H	
uL10m	Q9LHH1	At3g12370	GWHPDUBS004443	I	L7/L12 stalk
uL11m	Q9SVW7	At4g35490	GWHPBJSH065595	J	L7/L12 stalk
uL13m	Q7XA68	At3g01790	GWHPBJSH029441	K	
uL14m	Q93Z17	At5g46160	GWHPBJSH089764	L	
uL15m	Q9FLF3	At5g64670	GWHPBJSH025994	M	
uL16m	Q95747	AtMg00080	GWHPBJSH064047	N	
bL17m	Q8LD39	At5g09770	GWHPBJSH087064	O	
uL18m	Q8LDK5	At5g27820	GWHPBJSH011272 (A0A0D3DCH7)	P	Central Protuberance
bL19m	Q9T0D0	At4g11630	GWHPBJSH034513	Q	
bL20m	Q8LCN1	At1g16740	GWHPDUBS041804	R	
bL21m	Q8L9A0	At4g30930	GWHPBJSH021302 (Q8L9A0)	S	
uL22m	Q8LDU0	At4g28355	GWHPDUBS016275 (A0A0D3BFS0)	T	
uL23m	Q9SMR5	At4g39880	GWHPBJSH001034	U	
uL24m	Q9LT09	At5g23535	GWHPBJSH068111	V	
bL25-2m	Q9FKZ4	At5g66860	GWHPBJSH070969	W	Remodeled Domain III
bL25m	Q9SUR4	At4g23620	GWHPBJSH022473	X	
bL27m	Q94AC6	At5g15220	GWHPBJSH034173	Y	Central Protuberance
bL28m	Q9SV23	At4g31460	GWHPBJSH021367	Z	
uL29m	Q94JQ7	At1g07830	GWHPDUBS024649	a	
uL30m	Q8L908	At5g55140	GWHPBJSH092856	b	
bL31m	Q1G3G5	At5g55125	GWHPBJSH092851	c	Central Protuberance
bL32m	Q944L5	At1g26740	GWHPBJSH071754	d	
bL33m	Q9SQT5	At3g06320	GWHPDUBS049973	e	Central Protuberance
bL34m	Q84R26	At3g13882	GWHPBJSH048407	f	
bL35m	Q8LAA7	At5g45590	GWHPBJSH013115	g	
bL36m	Q8W464	At5g20180	GWHPDUBS011797 (Q8W464)	h	
mL40	Q9M0V5	At4g05400	GWHPDUBS047948	i	Central Protuberance
mL41	Q9LUJ9	At5g40080	GWHPDUBS013782	j	
mL43	Q9M1A3	At3g59650	GWHPDUBS043571	k	
mL46	Q8L7U3	At1g14620	GWHPBJSH030844	l	Central Protuberance
mL53	Q940G3	At5g39600	GWHPDUBS021933	m	L7/L12 stalk
mL54	Q9S799	At3g01740	GWHPBJSH029436	o	L7/L12 stalk
mL59/mL64	O65448	At4g22000	GWHPBJSH003404	p	Central Protuberance
mL60	Q94F28	At1g27435	GWHPDUBS017065	q	
mL80	Q9C9B5	At1g73940	GWHPDUBS033403	r	
mL87	Q9SD44	At3g51010	GWHPBJSH067819	s	
mL101 (rPPR4)	O22714	At1g60770	GWHPBJSH088794	t	Remodeled Domain III
mL102 (rPPR5)	Q9ZUU3	At2g37230	GWHPBJSH031757	u	
mL104 (rPPR9)	Q9FME4	At5g60960	GWHPBJSH068020	v	Remodeled Domain III

Supplementary Table 2 : List of the LSU r-proteins

List of the r-proteins found in the LSU. Proteins are colored by conservation with the bacterial ribosome (blue) other mitoribosomes (yellow) or specific to the plant mitoribosome (red). Protein modeled in the density are indicated. For proteins with UNIPROT IDs in parentheses, these were a better match than the botrytis sequences and were thus used instead.

Name of the protein	Arabidopsis UNIPROT	TAIR ID Arabidopsis	Modeled protein (Brassica oleracea var. botrytis)	ChainID	Location
uS2m	Q9GCB9	At3g03600	GWHPBJSH046906	A	body
uS3m	Q95749	AtMg00090	GWHPBJSH000741	B	head
uS4m	Q31708	AtMg00290	GWHPBJSH000696	C	body
uS5m	Q6GKU7	At1g64880	GWHPBJSH015007	D	body
bS6m	Q9LSA1	At3g18760	GWHPBJSH049377 (A0A0D3CIR5)	E	body
uS7m	P92557	AtMg01270	GWHPBJSH000704	F	head
uS8m	Q9M0E0	At4g29430	GWHPBJSH002412	G	body
uS9m	Q8L6Z4	At3g49080	GWHPBJSH080139	H	head and body
uS10m	P42797	At3g22300	GWHPBJSH050210	I	head
uS11m	Q8VZT8	At1g31817	GWHPBJSH051931	J	body
uS12m	P92532	AtMg00980	GWHPDUBS019876	K	body
uS13m	Q9CA19	At1g77750	GWHPBJSH064799	L	head
uS14m	Q9SMX4	At2g34520	GWHPDUBS019475	M	head
uS15m	Q9M8M9	At1g80620	GWHPBJSH065138	N	body
bS16m	Q9LTS6	At5g56940	GWHPBJSH018119	O	body
uS17m	Q9LHN1	At3g18880	GWHPBJSH049400	P	body
bS18m	Q9LML3	At1g07210	GWHPBJSH076274	Q	body
uS19m	P39697	At5g47320	GWHPBJSH072705 (P39697)	R	head
bS21m	F4JCI2	At3g26360	GWHPBJSH012122	S	body
bTHXm	Q9SJU8	At2g21290	GWHPDUBS022287	T	head
mS23	F4HPB7	At1g26750	GWHPBJSH071753	U	body
mS26	Q945P2	At5g49210	GWHPBJSH068774	V	body
mS29	Q8W4K2	At1g16870	GWHPBJSH054439	W	head
mS31/mS46	Q9C8L9	At1g53645	GWHPBJSH057126	X	head
mS33	Q8GXH5	At5g44710	GWHPDUBS036572	Y	head
mS34	Q9FHC3	At5g52370	GWHPBJSH018393	Z	body
mS35	Q9LJQ6	At3g18240	GWHPBJSH049245	a	head
mS37	A8MRX3	At1g47278	GWHPDUBS040247 (A8MRX3)	b	head
mS38	Q9FMK8	At5g63150	GWHPDUBS042575	c	body
mS41	Q6IDB8	At5g26800	GWHPBJSH068514	d	body
mS45	Q9LVA9	At5g62270	GWHPBJSH025481	e	body
mS47	Q8RXN4	At4g31810	GWHPBJSH021410	f	body
mS80 (rPPR6)	P0C896	At3g02650	GWHPBJSH010090 (P0C896)	l	head extension
mS83 (rPPR10)	Q1JPP0	At4g15640	GWHPBJSH050066	h	foot
mS77 (NFD5)	A0A654ECT9	At1g19520	GWHPBJSH053995 (A0A654ECT9)	i	body
mS76 (rPPR1)	Q8LE47	At1g61870	GWHPDUBS003368	j	foot
mS86	Q9FZ84	At1g18630	GWHPBJSH080930	k	body
mS81 (rPPR8)	Q8LPF1	At5g15980	GWHPBJSH095222 (Q8LPF1)	g	head extension
RsgA	Q4V399	At1g67440	GWHPBJSH063220	7	

Supplementary Table 3 : List of the SSU r-proteins

List of the r-proteins found in the SSU. Proteins are colored by conservation with the bacterial ribosome (blue) other mitoribosomes (yellow) or specific to the plant mitoribosome (red). Protein modeled in the density are indicated. For proteins with UNIPROT IDs in parentheses, these were a better match than the botrytis sequences and were thus used instead.

	Bacteria	Brassica oleracea var. botrytis				Helix	Domain	Putative enzyme (*A. thaliana*	Role
	Residue	Residue (Bo numbering)	Structure	Nanopore	Mass Spec				
SSU		A521 unknown	NO	YES	NO	h18	5'		
	m^{7}G527	**m^{7}G525**	YES	YES	YES	h18	5'	F4K7J0/AT5G50110	decoding center
		G527 unknown	NO	YES	NO	h18	5'		
	m^{2}G966	A959 unknown	NO	YES	NO	h31	3'M		
		**(m)A961**	NO	YES	YES	h31	3'M		
	m^{4}Cm1402	**m^{4}Cm1664**	YES	NO	YES	h44 (base)	3'm	Q9LEU9/AT5G10910	interacts with mRNA
		(m)C1671	NO	NO	YES	h44 (base)	3'm		
		U1804 unknown	NO	YES	NO	h44 (base)	3'm		
	m^{3}U1498	**m^{3}U1807**	YES	YES	YES	h44 (h44-h45 linker	3'm	Q6E297/At1g50000	shapes mRNA channel
	m^{2}G1516	m^{2}G1825	YES	NO	NO	h45	3'm		supports P-site conformation
	m$^6_2$A1518	**m$^6_2$A1827**	YES	YES	YES	h45	3'm	DIM1B Q9FK02/At5g66360	supports P-site conformation
	m$^6_2$A1519	**m$^6_2$A1828**	YES	NO	YES	h45	3'm	DIM1B Q9FK02/At5g66360	supports P-site conformation
LSU	Ψ746	Ψ891	YES	NO	NO	h35	II		
	Ψ955	Ψ1105	YES	NO	NO	h39	II		
		**Ψ2188**	YES	YES	YES	h68	IV		
		Ψ2191	N/A	NO	YES	h68	IV		
		A2227	N/A	YES	N/A	h69	IV		
	Ψ1911	**Ψ2229**	N/A	YES	YES	h69	IV		participates in the intersubunit bridge between h44 and domain IV
		A2231	N/A	YES	NO	h69	IV		
	m^{3}Ψ1915	**Ψ2233**	N/A	YES	YES	h69	IV		participates in the intersubunit bridge between h44 and domain IV, extends stacking and faces h44 of the small subunit
	Ψ1917	**Ψ2235**	N/A	YES	YES	h69	IV		participates in the intersubunit bridge between h44 and domain IV, extends stacking and faces h44 of the small subunit
		A2237	N/A	YES	NO	h69	IV		
		**Ψ2239**	N/A	YES	YES (mU)	h69	IV		
	m^{5}U1939	**m^{5}U2257**	YES	NO	YES	h70	IV	Q9LU28/AT3G21300	
	Gm2251	**Gm2560**	YES	YES	N/A	h80	V	Q8W497/AT2G19870	P-loop, G2560 is a key residue which base-pairs with the CCA-end of the P-site tRNA
		G2749	NO	YES	NO	h75	V		
	D2449	**D2754**	YES	YES	YES	ssRNA prior to h89	V		
	Ψ2457	Ψ2762	YES	NO	NO	h89	V		
	Cm2498	Cm2803	YES	NO	NO	h89	V		
	ho^{5}C2501	ho^{5}C2806	NO	YES	NO	ssRNA prior to h90	V		
	m^{2}A2503	**m^{2}A2808**	YES	YES	YES	ssRNA prior to h90	V	O80746/At1g60230	Peptide tunnel
	Ψ2504	**Ψ2809**	N/A	YES	YES	ssRNA prior to h90	V		
	Um2552	**Um2857**	YES	YES	YES	h92	V		A-loop, Intercalates between the bases of U2857 and G2858 (G2858 is a key residue which base-pairs with the CCA-end of the A-site tRNA)
		Gm2858	NO	YES	NO	h92	V		A-loop, G2858 is a key residue which base-pairs with the CCA-end of the A-site tRNA - The type of modification was assigned according to the human mitoribosome
		Ψ2860	NO	YES	NO	h92	V		
	Ψ2580	**Ψ2885**	YES	YES	YES	h90	V	PUS4 Q9LT72/At3g19440	stabilizes A-site tRNA
		G2886	NO	YES	NO	h90	V		
		Ψ2890	N/A	NO	YES	ssRNA prior to h93	V		
	Ψ2605	**Ψ2910**	YES	YES	N/A	h93	V		

<u>Supplementary Table 4:</u> List of the rRNA modifications

Summary of the rRNA modifications detected in B. oleracea mitoribosome. B. oleracea contains 11 modified residues in the SSU and 19 in the LSU. The method of detection (Cryo-EM structure, Mass spectrometry or Nanopore DRS), their helix and domain position on the rRNA, as well as their functional role are presented for each modified residue.

Oligonucleotide	Sequence	Method
26S_F	CGAAAAGAATGCATTGGAT	Nanopore
26S_R	TCGTTTAGTACGAGATGGC	Nanopore
18S_F	ATCATAGTCAAAAGAAGAGTTTG	Nanopore
18S_R	GGATTCAATCCAGCCACAG	Nanopore
5S_F	AAACCGGGCACTACGG	Nanopore
5S_R	TTCACCGGGCTTAGACC	Nanopore
T7-26S	TAATACGACTCACTATAGCGAAAAGAATGCATTGGAT	Nanopore
T7-18S	TAATACGACTCACTATAGATCATAGTCAAAAGAAGAGTTTG	Nanopore
T7-5S	TAATACGACTCACTATAGAAACCGGGCACTACGG	Nanopore
RTA_OligoA_BC1	/5PHOS/GGCTTCTTCTTGCTCTTAGGTAGTAGGTTC	Nanopore
RTA_OligoB_26S_BC1	GAGGCGAGCGGTCAATTTTCCTAAGAGCAAGAAGAAGCCTCGTTTAGTA	Nanopore
RTA_OligoB_18S_BC1	GAGGCGAGCGGTCAATTTTCCTAAGAGCAAGAAGAAGCCGGATTCAATC	Nanopore
RTA_OligoB_5S_BC1	GAGGCGAGCGGTCAATTTTCCTAAGAGCAAGAAGAAGCCTTCACCGGGC	Nanopore
RTA_OligoA_BC3	/5PHOS/GTACTTTTCTCTTTGCGCGGTAGTAGGTTC	Nanopore
RTA_OligoB_26S_BC3	GAGGCGAGCGGTCAATTTTCCGCGCAAAGAGAAAAGTACTCGTTTAGTA	Nanopore
RTA_OligoA_BC2	/5PHOS/GTGATTCTCGTCTTTCTGCGTAGTAGGTTC	Nanopore
RTA_OligoB_18S_BC2	GAGGCGAGCGGTCAATTTTCGCAGAAAGACGAGAATCACGGATTCAATC	Nanopore
RTA_OligoA_BC4	/5PHOS/GGTCTTCGCTCGGTCTTATTTAGTAGGTTC	Nanopore
RTA_OligoB_5S_BC4	GAGGCGAGCGGTCAATTTTAATAAGACCGAGCGAAGACCTTCACCGGGC	Nanopore
Barcode 1	GGCTTCTTCTTGCTCTTAGG	Nanopore
Barcode 2	GTGATTCTCGTCTTTCTGCG	Nanopore
Barcode 3	GTACTTTTCTCTTTGCGCGG	Nanopore
Barcode 4	GGTCTTCGCTCGGTCTTATT	Nanopore
18S_1	CATGTCATGATCGCGCACTCG	MS
18S_2	CCGAAGAACACTTGCCCCCCC	MS
18S_3	CACCGCTTGTGCAGGCCCCCG	MS
18S_4	TAAGGATAGGGTTTCTCGTTC	MS
18S_5	GTGTGTACAGGGCCCGGGTAC	MS
18S_6	ACACAGAAGTGCTGGGTGATC	MS
26S_1	CGTGGGTTCGGTCCTCCAAGG	MS
26S_2	ACCAGCTATATCCGATCTTGG	MS
26S_3	GTCTGTCTGTACAAACGAGA	MS
26S_4	CTAAGTGATTGCTTAGGGACC	MS
26S_5	CTTTCCAGCACCGGGCAGGTG	MS
26S_6	GCAGTCGTTACACCATTCGTG	MS
26S_7	CCCGGCGTACCTTTGATCCGT	MS
26S_8	GGACCTTCTTCAACCCCAGGA	MS
26S_9	TAACACCAACGGTAGATAGGA	MS

Supplementary Table 5 : List of oligonucleotides

FLO-PRO002 run	Barcode ID	Target rRNA	direct RNA-seq reads available
TGS104_16052024	Barcode01	Native 26S	17 849
TGS104_14052024	Barcode03	IVT 26S	680 928
TGS103_21032024	Barcode01	Native 18S	2 463 135
TGS103_21032024	Barcode02	IVT 18S	1 587 154
**FLO-PRO002 run**	**Barode ID**	**Target rRNA**	**Selected full-length reads**
TGS104_16052024	Barcode01	Native 26S	2166
TGS104_14052024	Barcode03	IVT 26S	2190
TGS103_21032024	Barcode01	Native 18S	2000
TGS103_21032024	Barcode02	IVT 18S	2000

Supplementary Table 6 : Read statistics from Deeplexicon demultiplexing, length filtering and read selection

	Position of Interest	Nucleotide	Reference Nucleotide	Error IVT (%)	Error Native (%)	Δcallerror (%ErrorNat-%ErrorIVT)
18S	**521**	G	A	2,25	18,55	16,3
	**525**	C	G	0,21	23,39	23,18
	**527**	A	G	4,04	29,81	25,77
	**959**	C	A	3,02	33,38	30,36
	**961**	C	A	0,97	23,28	22,31
	**1664**	U	C	4,4	2,02	-2,38
	**1671**	U	C	2,9	4,51	1,61
	**1804**	A	U	1,19	22,96	21,77
	**1807**	C	U	1,52	14,12	12,6
	**1825**	C	G	0,36	2,73	2,37
	**1827**	C	A	0	13,11	13,11
	**1828**	U	A	1,83	8,34	6,51
26S	**891**	C	U	1,49	1,61	0,12
	**1105**	C	U	1,52	1,98	0,46
	**2188**	C	U	0,6	53,31	52,71
	**2191**	A	U	1,07	2,71	1,64
	**2227**	G	A	2,25	18,85	16,6
	**2229**	C	U	0,28	34,63	34,35
	**2231**	U	A	3,93	15,96	12,03
	**2233**	C	U	0,33	44,68	44,35
	**2235**	C	U	0,91	68,35	67,44
	**2237**	C	A	0,09	13,41	13,32
	**2239**	C	U	0,28	47,63	47,35
	**2257**	C	U	2,53	5,6	3,07
	**2560**	U	G	0,89	13,91	13,02
	**2749**	A	G	13,2	30,13	16,93
	**2752**	U	G	0,47	21,6	21,13
	**2753**	C	A	0,24	27,03	26,79
	**2754**	C	U	1,2	27,12	25,92
	**2755**	C	A	0,82	19,72	18,9
	**2756**	G	A	0,56	14,5	13,94
	**2762**	A	U	0,14	0,52	0,38
	**2803**	U	C	0,46	0,65	0,19
	**2806**	U	C	6,57	39,63	33,06
	**2808**	U	A	0,14	38,01	37,87
	**2809**	C	U	3,17	46,36	43,19
	**2857**	C	U	0,64	39,9	39,26
	**2858**	C	G	0,23	13,9	13,67
	**2860**	C	U	0,51	18,83	18,32
	**2885**	C	U	0,46	41,42	40,96
	**2886**	A	G	2,3	32,12	29,82
	**2890**	C	U	2,61	2,78	0,17
	**2910**	C	U	2,01	94,32	92,31

Supplementary Table 7: Base-calling errors of positions of interest
