# Supplementary material for: Structural insights into cauliflower mitoribosome in translation state and in association with a late assembly factor
Source: Nat Commun. 2025 Dec 2;16:10839. doi: 10.1038/s41467-025-65864-z (PMC12672651; doi:10.1038/s41467-025-65864-z)
Supplement: Supplementary file 2 — Description of Additional Supplementary Files [file 41467_2025_65864_MOESM2_ESM.pdf]

Description of Additional Supplementary Files

File name: Supplementary Movie 1

Description: Result of the cryoSPARC 3D Flexible refinement. The 4 main different modes of motion computed by the cryoSPARC 3D Flexible refinement are shown.
