# Supplementary material for: Structural insights into cauliflower mitoribosome in translation state and in association with a late assembly factor
Source: Nat Commun. 2025 Dec 2;16:10839. doi: 10.1038/s41467-025-65864-z (PMC12672651; doi:10.1038/s41467-025-65864-z)
Supplement: Supplementary file 4 — Reporting Summary [file 41467_2025_65864_MOESM4_ESM.pdf]

nature portfolio

Corresponding author(s): *Double-anonymous peer review submissions: write DAPR and your manuscript number here instead of author names.*

Last updated by author(s): Sep 4, 2025

Reporting Summary

Nature Portfolio wishes to improve the reproducibility of the work that we publish. This form provides structure for consistency and transparency in reporting. For further information on Nature Portfolio policies, see our Editorial Policies and the Editorial Policy Checklist.

Statistics

For all statistical analyses, confirm that the following items are present in the figure legend, table legend, main text, or Methods section.

n/a	Confirmed	
☐	☒	The exact sample size (*n*) for each experimental group/condition, given as a discrete number and unit of measurement
☐	☒	A statement on whether measurements were taken from distinct samples or whether the same sample was measured repeatedly
☐	☒	The statistical test(s) used AND whether they are one- or two-sided<br>*Only common tests should be described solely by name; describe more complex techniques in the Methods section.*
☒	☐	A description of all covariates tested
☐	☒	A description of any assumptions or corrections, such as tests of normality and adjustment for multiple comparisons
☐	☒	A full description of the statistical parameters including central tendency (e.g. means) or other basic estimates (e.g. regression coefficient) AND variation (e.g. standard deviation) or associated estimates of uncertainty (e.g. confidence intervals)
☐	☒	For null hypothesis testing, the test statistic (e.g. *F*, *t*, *r*) with confidence intervals, effect sizes, degrees of freedom and *P* value noted<br>*Give P values as exact values whenever suitable.*
☒	☐	For Bayesian analysis, information on the choice of priors and Markov chain Monte Carlo settings
☒	☐	For hierarchical and complex designs, identification of the appropriate level for tests and full reporting of outcomes
☒	☐	Estimates of effect sizes (e.g. Cohen's *d*, Pearson's *r*), indicating how they were calculated

Our web collection on statistics for biologists contains articles on many of the points above.

Software and code

Policy information about availability of computer code

Data collection	Cryo-EM data were collected using a 300kV G4 Titan Krios electron microscope (ThermoScientific) equipped with Falcon4i camera and a Selectris X energy filter using EPU for automated data acquisition.<br>The Oxford Nanopore Direct RNA Sequencing (DRS) runs were performed on PromethION FLO-PRO002 R9.4.1 flowcells.<br>MS and tandem MS analyses of rRNA modifications were performed using a SYNAPT G2-S (Waters Corporation).
Data analysis	For cryo-EM data processing: cryoSPARC (v4.4), ChimeraX (v1.8), COOT (v0.8.9), PHENIX (v1.21)<br>For Oxford Nanopore DRS analysis: Deeplexicon (v1.2.0), Guppy (v6.3.8), minimap2 (v2.26), f5c (v1.5), Rsamtools (v2.20.0), ggplot2 (v3.5.1) and custom R scripts provided at https://doi.org/10.5281/zenodo.14196969.

For manuscripts utilizing custom algorithms or software that are central to the research but not yet described in published literature, software must be made available to editors and reviewers. We strongly encourage code deposition in a community repository (e.g. GitHub). See the Nature Portfolio guidelines for submitting code & software for further information.

Data

Policy information about availability of data

All manuscripts must include a data availability statement. This statement should provide the following information, where applicable:

- Accession codes, unique identifiers, or web links for publicly available datasets
- A description of any restrictions on data availability
- For clinical datasets or third party data, please ensure that the statement adheres to our policy

The single particle cryo-EM maps of B. oleracea mitoribosome have been deposited at the Electron Microscopy Data Bank (EMDB) and models on the protein data bank (PDB). Full high-resolution mitoribosome EMD-51718 (PDB 9GYT), unfocused EMD-51703, focused LSU EMD-51710, focused LSU CP EMD-51711, focused LSU L7/12 stalk EMD-51712, focused LSU back extension EMD-51713, focused LSU rPP5 EMD-51714, focused SSU body EMD-51704, focused SSU body protuberance EMD-51709, focused SSU foot rPPR1 EMD-51708, focused SSU foot rPPR10 EMD-51707, focused SSU head EMD-51705, focused SSU head S3 area EMD-51706, focused SSU head extension base EMD-51715, focused SSU head extension core EMD-51716, focused SSU head extension tip EMD-51717. Small subunit in presence of RsgA EMD-50014 (PDB 9EVT), focused SSU head EMD-50015, focused SSU body EMD-50017, focused RsgA EMD-50016. P-site tRNA mitoribosome stalled with chloramphenicol EMD-50011 (PDB 9EVS), focused LSU EMD-50012, focused SSU EMD-50013.
The Oxford Nanopore DRS datasets and bioinformatics code for the detection of rRNA modifications are available at the Zenodo database: https://doi.org/10.5281/zenodo.14196969.
MS/MS spectra used to identify rRNA modifications are provided in Supplementary Figure 11.

Research involving human participants, their data, or biological material

Policy information about studies with human participants or human data. See also policy information about sex, gender (identity/presentation), and sexual orientation and race, ethnicity and racism.

Reporting on sex and gender	n/a
Reporting on race, ethnicity, or other socially relevant groupings	n/a
Population characteristics	n/a
Recruitment	n/a
Ethics oversight	n/a

Note that full information on the approval of the study protocol must also be provided in the manuscript.

Field-specific reporting

Please select the one below that is the best fit for your research. If you are not sure, read the appropriate sections before making your selection.

☒ Life sciences ☐ Behavioural & social sciences ☐ Ecological, evolutionary & environmental sciences

For a reference copy of the document with all sections, see nature.com/documents/nr-reporting-summary-flat.pdf

Life sciences study design

All studies must disclose on these points even when the disclosure is negative.

Sample size	For cryo-EM analysis, one biological sample were analysed for each of the conditions, which resulted in the "high-resolution" mitoribosome, the stalled mitoribosome, and the SSU in presence of RsgA.<br>For Oxford Nanopore DRS analysis, one biological sample was analyzed for the native rRNA transcripts. Two additional samples were analyzed for the in vitro transcribed (IVT) transcripts of 26S and 18S.<br>For MS analyses, biological samples were analysed for native rRNAs from 3 independent rRNA preparations.
Data exclusions	No data were excluded.
Replication	rRNA base modifications were detected using four Oxford Nanopore DRS data subsets:<br>1. 2000 single-molecule reads of native 18S rRNA<br>2. 2000 single-molecule reads of IVT 18S rRNA<br>3. 2166 single-molecule reads of native 26S rRNA<br>4. 2190 single-molecule reads of IVT 26S rRNA
Randomization	No assays were conducted that required randomization.

Blinding	Blinding was not relevant to this study as all analyses were based on mitochondrial ribosome purification, RNA sequencing, MS analyses and structural analysis of cryo-EM data from RNA and proteins, and thus were not prone to individual/subjective biases.

Reporting for specific materials, systems and methods

We require information from authors about some types of materials, experimental systems and methods used in many studies. Here, indicate whether each material, system or method listed is relevant to your study. If you are not sure if a list item applies to your research, read the appropriate section before selecting a response.

Materials & experimental systems

n/a	Involved in the study	
☒	☐	Antibodies
☒	☐	Eukaryotic cell lines
☒	☐	Palaeontology and archaeology
☒	☐	Animals and other organisms
☒	☐	Clinical data
☒	☐	Dual use research of concern
☐	☒	Plants

Methods

n/a	Involved in the study	
☒	☐	ChIP-seq
☒	☐	Flow cytometry
☒	☐	MRI-based neuroimaging

Dual use research of concern

Policy information about dual use research of concern

Hazards

Could the accidental, deliberate or reckless misuse of agents or technologies generated in the work, or the application of information presented in the manuscript, pose a threat to:

No	Yes	
☒	☐	Public health
☒	☐	National security
☒	☐	Crops and/or livestock
☒	☐	Ecosystems
☒	☐	Any other significant area

Experiments of concern

Does the work involve any of these experiments of concern:

No	Yes	
☒	☐	Demonstrate how to render a vaccine ineffective
☒	☐	Confer resistance to therapeutically useful antibiotics or antiviral agents
☒	☐	Enhance the virulence of a pathogen or render a nonpathogen virulent
☒	☐	Increase transmissibility of a pathogen
☒	☐	Alter the host range of a pathogen
☒	☐	Enable evasion of diagnostic/detection modalities
☒	☐	Enable the weaponization of a biological agent or toxin
☒	☐	Any other potentially harmful combination of experiments and agents

Plants

Seed stocks	n/a plant material was purchased from the local supermarket
Novel plant genotypes	n/a
Authentication	n/a
